# S01 A Systems-based Approach to Physical Activity in Scotland

**DOI:** 10.1093/eurpub/ckae114.196

**Published:** 2024-09-26

**Authors:** Marie Murphy

**Affiliations:** University of Edinburgh

## Abstract

**Purpose:**

To share learning from the development and application of a pragmatic, evidence-driven, systems-based approach to physical activity policy in Scotland at a national and local level.

**Description:**

Systems-based approaches are increasingly used to respond to complex public health issues such as physical inactivity. Public Health Scotland (PHS) developed an evidence-driven, yet pragmatic process, to enable policy makers and practitioners to implement a systems-based approach to Physical Activity (PA) in Scotland.

The process was informed by and adapted from systems-based thinking underpinning public health reform in Scotland and a whole system approach to obesity in England and draws on methodologies familiar to policymakers and practitioners such as the quality improvement and outcome focused planning.

PHS drew on existing evidence and learning to translate the scientific evidence of what works to increase population levels of PA into a Scottish context. This informed the development of eight strategic delivery outcomes and a logic model, accompanied by a 5-stage process used to inform national and local collaborative strategic actions.

Nationally, adopted by Scottish Government and partners to guide the development of a cross-policy Physical Activity for Health Strategy for Scotland and to update the Active Scotland Outcomes Framework, indicators and target.

Locally, PHS and sportscotland (the national agency for sport) work collaboratively with local authorities and their community planning partners to apply the approach to inform local Physical Activity Strategies and Action Plans. Increasing the breadth of policy partners involved; local government, transport, planning, education, health, social care and sport and leisure, reflecting a system wide approach to PA.

Implementation is monitored via governance structures, national surveillance indicators, process indicators and an ongoing cycle of quality improvement and policy review.

Transferable across national and local government this approach has positively impacted on the development of national and local evidence-based PA policy, enabling partners to maximise existing resources, whilst contributing to PA outcomes and their respective policy outcomes.

